# A Study on the Development of Information and Communication Technology-Based Oral Functional Rehabilitation Exercise Program Content for Elderly People

**DOI:** 10.3390/healthcare12202058

**Published:** 2024-10-16

**Authors:** Yong-Keum Choi, Ji-Hye Yun, Hyun Lee, Eun-Gyeong Cha, Hyang-Ah Park

**Affiliations:** 1 Department of Dental Hygiene, College of Health Science, SunMoon University, Asan 31460, Republic of Korea; cherishgold@hanmail.net; 2 Genome-Based BioIT Convergence Institute, SunMoon University, Asan 31460, Republic of Korea; 3 Division of Population Health Research, Department of Precision Medicine, Korea National Institute of Health, Korea Disease Control and Prevention Agency (KDCA), Cheongju 28159, Republic of Korea; dream10307@naver.com; 4 Department of Computer Science, SunMoon University, Asan 31460, Republic of Korea; mahyun91@sunmoon.ac.kr; 5 Department of Preventive and Social Dentistry, College of Dentistry, Kyung Hee University, Seoul 02447, Republic of Korea; cha_ek@naver.com

**Keywords:** digital health, elderly, exercise, oral health, rehabilitation

## Abstract

**Background/Objectives**: This study was conducted to develop information and communication technology (ICT)-based oral functional rehabilitation exercise (OFRE) program content to effectively improve the oral function of the elderly people. **Methods**: After selecting evidence-based effective OFRE items through systematic review, the final items were constructed through the validity evaluation of detailed items through an expert Delphi survey. The items were composed in a simple content form that can be performed directly and applied to ICT-based mobile applications. **Results**: The final content items consisted of an oral functional motor-ability measurement, oral Pilates videos, and games. The first is to measure the maximum opening amount before and after exercise, and the opening amount was designed to be measured by eating the fruit displayed on the screen by opening and closing the lips. The second one consisted of eight exercises in the video, and each exercise was to be performed at least three times a day, with a total of two sets. The third is a salivary secretion function exercise that stimulates the salivary glands to stimulate the user’s interest and enable them to perform oral movements on their own. It consists of a lip and respiratory muscle exercise that inflates the cheeks and bursts a balloon, and the image disappears when the word in the image presented on the screen is pronounced correctly. It consists of pronunciation exercises. **Conclusions**: This content development attempt can be expanded into new convergence research linked to ICT and can be used as basic data when developing related content as part of digital care for the elderly in the future.

## 1. Introduction

As diverse problems due to aging emerge as national issues, advanced countries have emphasized the utilization of information and communication technology (ICT) to enhance the effectiveness of community welfare services and address social issues arising from aging [1]. For instance, the European Union actively promotes ICT-based elderly friendly R&D programs to support the “active and healthy aging” of the aging population [2]. Similarly, Japan is focusing on using intelligent information technologies, such as artificial intelligence and robotics, to improve the quality and productivity of services in healthcare, medical, and caregiving fields, aiming to enhance the quality of life for the elderly [3].

These developments signal a paradigm shift in elderly healthcare from disease-centric services to preventive and proactive management services [4], reflecting an increasing demand for wellness technologies focused on improving the quality of life for elderly individuals [5]. Consequently, many countries are actively researching ICT-based elderly health management to prevent functional decline and manage diverse functional statuses in elderly people [6,7,8]. In the medical field, for example, ICT-based rehabilitation exercise programs are being actively developed. Kim et al. developed ICT-based exercise rehabilitation services that facilitated accurate and safe exercise participation for patients requiring rehabilitation within the local community, as well as advanced system development to strengthen linkages between hospitals and local areas [9]. Furthermore, research is exploring various aspects of the linkage between ICT and medical services, such as developing individualized cognitive training systems based on ICT for elderly individuals in dementia facilities [10] and ICT-based home-care cardiac rehabilitation systems for elderly patients with heart failure [11].

In line with these trends, the field of dentistry is actively exploring ICT-based dental medical services. ICT-based content is being developed for orthodontic patients to provide relevant information and educational materials [12]. Additionally, ICT-based oral health education systems for elderly people have been developed to provide educational information aimed at improving oral health for seniors, demonstrating significant effectiveness [13].

Despite the development of diverse ICT-based oral care content, there is still a scarcity of ICT-based content developed for oral rehabilitation in the elderly. As aging inevitably leads to a decline in oral function, providing rehabilitation interventions to prevent and manage this decline is essential [14]. Many previous studies have developed oral exercise interventions aimed at rehabilitating oral function among elderly people [15,16,17]. In a study by Kim et al., improvements in occlusal force and masseter muscle thickness were observed in individuals aged 65 and older who performed oral-function rehabilitation exercises (OFRE) in the local community [18]. Additionally, Shirobe et al. demonstrated improvements in occlusal pressure and articulatory function through oral exercises, including warm-up exercises, mouth-opening exercises, tongue-pressure exercises, articulatory exercises, and oral exercise programs for elderly individuals residing in the local community [19]. The World Dental Federation (FDI) recently proposed guidelines for OFREs to prevent oral-function decline in elderly individuals, based on accumulated evidence of oral exercise [20]. While OFREs are gaining attention as an effective management method for oral-function rehabilitation in the elderly, they are often delivered through one-time lectures and education by service providers, highlighting a significant shortage of ICT-based content that can be applied daily and repeatedly. Additionally, there is a notable lack of studies that organize programs and content based on theoretical foundations in the current field of dentistry [21,22].

Therefore, this study organized oral-function rehabilitation-related items established based on evidence into three ICT-based programs and developed detailed content within these programs. Through this, we aimed to identify the potential for developing a program for the oral-function rehabilitation of the elderly and an effective method for content composition.

## 2. Materials and Methods

### 2.1. Study Model and Period

**Figure 1:**
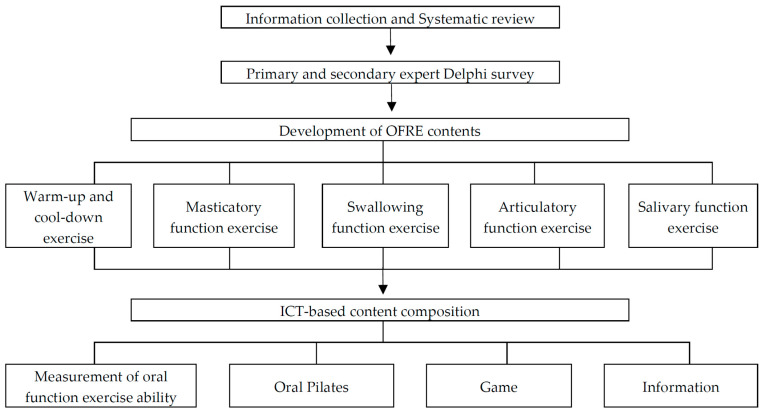
The development process of ICT-based OFRE content.

This study employed a mixed-methods approach, combining quantitative and qualitative methods, to develop an OFRE program and content for the elderly in facilities. The overall design can be described as a sequential explanatory design. The program items were selected based on the contents established through a systematic literature review and the Delphi survey, and the detailed contents were continuously revised and supplemented by three dental hygienists with more than five years of clinical experience and one professor of dental hygiene from March 2022 to December 2023. In addition, one professor of computer science and engineering assisted in the realization and application of ICT-based content. The flow chart of the entire development is as shown in Figure 1.

### 2.2. ICT-Based Content Development Process 1: OFRE App Content Survey

To identify the current status of mobile applications related to oral function, a keyword search was conducted on the Google Play Store (Google LLC, Mountain View, CA, USA) and Apple App Store (Apple Inc., Cupertino, CA, USA) for the month of July 2022 using the keywords ‘Oral health’, ‘Tongue therapy’, ‘Oral motor’, ‘Swallowing’, ‘Dysfagi’, and ‘Oral motor exercise’. A total of 10 related applications were retrieved, and the purpose, operating system, and contents of the applications were identified through the information provided in the store and downloads. As a result, ICT-based oral functional rehabilitation exercises are very rare, and the need for OFRE-centered content organization has been confirmed (Table 1).

### 2.3. Process 2: Evidence-Based OFRE Content Categorization through Systematic Review

A systematic review of the literature related to oral exercise interventions was conducted to organize ICT-based OFRE content for older adults. The PubMed, Ovid Embase, Cochrane Library, and CINAHL databases were searched up to 10 August 2022, and a total of 1007 articles were retrieved, excluding duplicates. After excluding 908 articles that did not meet the inclusion criteria through titles and abstracts, a total of 19 articles were selected through the screening process. The intervention areas of the studies were double-counted in consideration of the studies that studied multidisciplinary interventions, and finally, oral functions were categorized into mastication, swallowing, articulation, and salivation.

### 2.4. Process 3: Validity Evaluation of the Detailed OFRE Content through the Expert Delphi Survey

To determine the validity of the detailed content of the OFRE items derived through this systematic review, a panel of eight experts in geriatric dentistry and geriatric dental hygiene was formed, and a total of two revised expert Delphi surveys were conducted (IRB no. SM-202211-046-1) [23]. The second expert Delphi survey provided expert opinions on warm-up and cool-down exercise, masticatory function exercise, tongue exercise, pharyngeal exercise, lip and respiratory muscle exercise, articulatory function exercise, and salivary function exercise for oral-function rehabilitation in elderly people. The validity of the number and method of exercise(s) was confirmed through matching, and the detailed contents are shown in Table 2.

### 2.5. Process 4: Composition of the Final ICT-Based OFRE Contents

To confirm whether the derived detailed content could be applied as ICT-based content, advice was sought from a computer engineering professor, and the final content composition was completed. ICT-based content largely consists of oral functional exercise ability evaluation, oral Pilates, and games. Oral functional exercise ability is measured before and after the OFRE, and the maximum opening amount is measured. Oral Pilates consists of eight videos of the OFRE so that you can perform the exercise. Finally, the game was configured to perform OFRE based on motion recognition. The detailed contents of each are shown in Table 3.

## 3. Results

### 3.1. Content of ICT-Based OFRE for Measuring Oral Function Exercise Ability Using Facial Recognition

**Figure 2:**
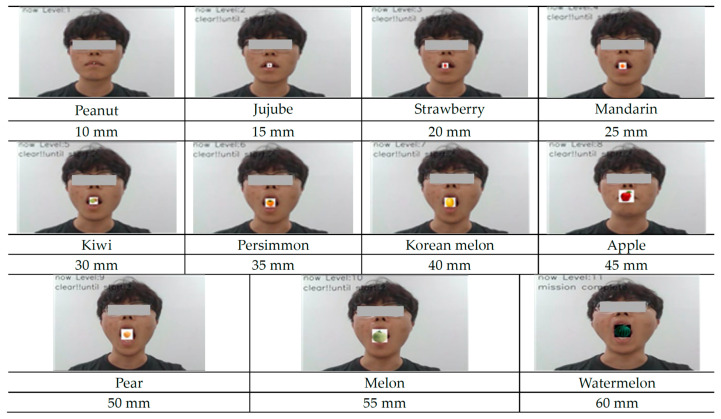
ICT-based OFRE content for measuring oral-function exercise ability using facial recognition.

Oral functional exercise ability was measured through the ability to recognize the user’s face on the screen through the front camera of the mobile device to measure the amount of mouth opening (Figure 2). The opening amount ranged from a minimum of 10 mm to a maximum of 60 mm based on the normal opening amount of 40 mm for adults, and the opening amount was divided into levels 1 to 11 and could be measured in 5 mm increments [24]. When measuring the opening amount, images of food of various sizes were obtained at each stage to make it more accessible and to stimulate user interest. Step by step, when the food image presented on the screen is placed in the mouth, the success of the opening is judged, and the next step is taken. It is designed to measure the user’s oral-function exercise ability through the maximum size of the food opening. The oral functional exercise ability test was designed to be performed before and after oral Pilates to measure the degree of change in mouth opening before and after exercise.

### 3.2. ICT-Based OFRE Oral Pilates Content

**Figure 3:**
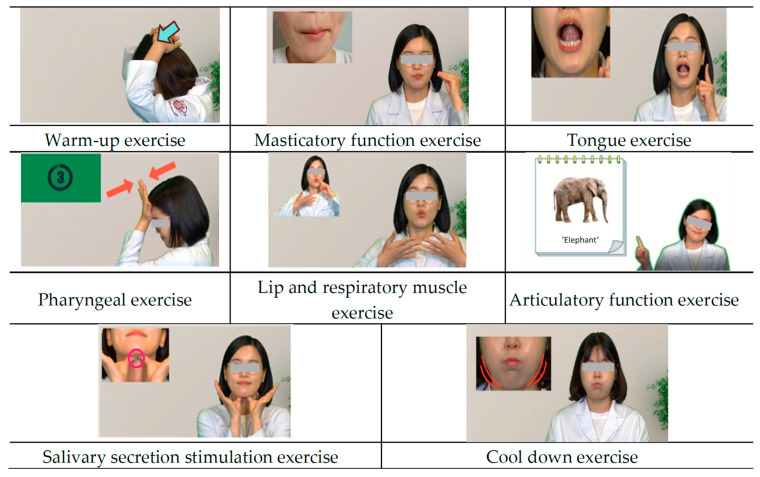
ICT-based content of OFRE Oral Pilates.

Oral Pilates produced a video of a total of eight exercises that users could watch and follow (Figure 3). Each video consists of a detailed description of the exercise to be performed, a video of the exercise being performed, and guidance on the next exercise to be performed. The detailed explanation of the exercise to be performed took into account the user’s age and level of understanding, and the exercise performance video was also designed to increase participation through an enlarged screen and the placement of icons to aid understanding. The break time between videos was set at 60 s to reduce muscle fatigue and maintain concentration. Oral Pilates consists of eight images performed three times each in one set and is designed to be performed at least two times per day.

### 3.3. ICT-Based OFRE Game Content

**Figure 4:**
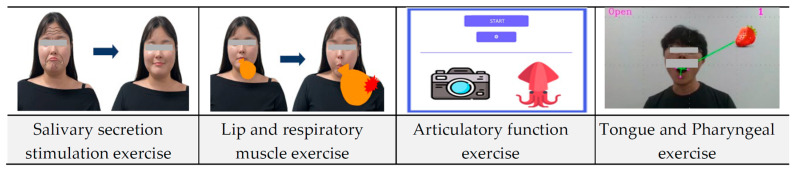
ICT-based OFRE game content.

This study developed four ICT-based games, utilizing oral Pilates exercises, to enhance engagement and promote sustained oral-function improvement (Figure 4). Each game employed facial recognition (front-facing camera) and voice recognition (microphone) technology to provide real-time feedback on user performance.

#### 3.3.1. Salivary Secretion Stimulation Game

- A virtual wrinkle pattern was displayed on the user’s facial image. As the user performed a salivary gland stimulation massage, the wrinkle pattern’s reduction was tracked in real-time. The complete disappearance of the wrinkles (100% reduction) indicated successful game completion. This method objectively assessed the accuracy and duration of the massage technique in stimulating salivary secretion.

#### 3.3.2. Lip and Respiratory Muscle Exercise Game

- Users virtually inflated an on-screen balloon by puffing out their cheeks. Balloon size was measured in pixels. The balloon’s inflation to a predetermined size (causing it to “burst”) marked successful game completion. This measured the intensity and duration of lip and respiratory muscle engagement.

#### 3.3.3. Articulatory Function Exercise Game

- Users pronounced words corresponding to images displayed on the screen. Voice recognition technology analyzed pronunciation accuracy and duration. Successful game completion was achieved upon the correct pronunciation of a predetermined number of words. This assessed the accuracy and speed of articulatory exercises.

#### 3.3.4. Tongue and Pharyngeal Exercise Game

- Using facial recognition, the accuracy of the user’s tongue contact with an on-screen target (simulating food) was measured. This provided an objective assessment of tongue and pharyngeal muscle function.

## 4. Discussion

Recently, with the rapid increase in the elderly population, interest in healthy old age has increased [25]. To live a healthy life, oral health must be considered along with physical and mental health [26]. In particular, the importance of oral function is being recognized for its correlation with systemic health and for elderly people’s active participation in social activities [27,28]. Accordingly, various attempts are being made to improve oral function. In particular, as the use of mobile devices by elderly people has steadily increased recently, various methods to improve oral function based on ICT have been explored [29,30,31]. Therefore, this study was conducted to provide the basic data needed to build an ICT-based oral functional rehabilitation exercise system by developing ICT-based oral functional rehabilitation exercise content to help elderly people lead healthy and active lives.

First, in this study, ICT-based measurements of oral functional exercise ability content were constructed to measure changes in mouth opening before and after oral Pilates exercises. In a study by Jang et al. [32,33], it was confirmed that the maximum amount of mouth opening increased when an oral exercise program was applied to elderly people, confirming that the amount of mouth opening can be used as an appropriate evaluation index for oral functional rehabilitation exercise. In addition, when measuring the amount of mouth opening by configuring fruit images according to the degree of mouth opening, the fruit can advance to the next level when the mouth opening is successful, thereby arousing interest and improving participation in the elderly [34]. This measurement of oral functional exercise ability before and after exercise allows elderly people to visually confirm the degree of change, and it is believed that this can lead to a motivation to participate in exercise and be used as effective content [35].

Second, in this study, a systematic review and expert Delphi survey were conducted to develop evidence-based oral functional rehabilitation exercises, and eight oral Pilates methods were developed. Oral exercises to improve oral function in elderly people have been developed in various ways. In a study by Ki et al. [36], oral motor content consisted of gum chewing, clock-sound pronunciation exercises, and salivary gland massage. The study by Ohara et al. [37] involved deep breathing, neck and oromaxillofacial stretching, and salivary gland massage. The oral Pilates test developed in this study classified the oral function of elderly individuals based on evidence and was organized according to function. It was composed of a warm-up exercise, main exercise, and cool-down exercise considering the oral muscle condition of the elderly individual. The appropriate number and duration of exercise(s) were also determined based on evidence obtained through a systematic review and an expert Delphi survey [23]. This evidence-based approach is different from previous oral exercise programs in that it does not target only specific oral functions of elderly people but can improve overall oral function and is designed to be performed safely and systematically. To date, a variety of oral exercise programs that can be performed face to face at senior welfare centers and sports facilities have been developed, but ICT-based oral exercise that can be easily followed at home is lacking. In this reality, ICT-based oral Pilates is meaningful because elderly people can easily perform it at home, and it is a form of content that is simply structured considering the digital literacy of elderly people to increase accessibility. In particular, as current elderly healthcare practices pursue the concept of Aging in Place, there is a need for research and development in visiting medical care, home healthcare, and gerontechnology that allows elderly people to receive services at home or in the community. Therefore, ICT-based oral Pilates can be said to be timely content suited to the times.

Third, in this study, eight OFREs were divided into salivary secretion stimulation exercises, lip and respiratory muscle exercises, articulatory function exercises, and tongue and pharyngeal exercises, and the games were composed of a total of four types. According to Sánchez-Mena et al., the gamification of learning provides enjoyment in learning activities and enhances concentration, making learning more accessible [38]. In addition, it can induce the voluntary and active participation of elderly people, making them perceive learning activities as a fun game process and promoting learning by arousing interest and providing continuous motivation [39]. Based on these characteristics, education gamification is spreading to various fields, such as the medical, health, and education fields. It has also been applied to offline education in traditional classrooms and online learning content and is actively used both online and offline [40]. Accordingly, in this study, the gamification of oral functional rehabilitation exercise content was designed to increase participation by increasing the interest of elderly people and promoting continuous exercise performance. It is believed that this will help elderly people continue to perform oral functional rehabilitation exercises on their own. However, it is difficult to measure the effectiveness of oral functional rehabilitation exercises through games at the planning stage. A game is designed based on facial recognition and voice recognition, so there is a limitation in that equipment with camera and microphone functions, such as a computer, tablet, or smartphone, is required to play the game. Accordingly, it is necessary to confirm the effectiveness of oral functional rehabilitation exercise games through the development and commercialization of planned content and active clinical research, and it will be necessary to develop them into a form that has fewer equipment restrictions and can be easily performed.

The limitations of this study are as follows. First, while the current study demonstrated the potential of the developed program and content, further research is needed to assess its long-term effectiveness and broader applicability in diverse elderly populations. This future research will include a two-phased approach: a pilot study to evaluate usability and initial efficacy with a small sample of older adults, followed by a larger-scale longitudinal clinical trial focusing on improved oral function, improved quality of life, etc. Second, it was not confirmed whether the ICT-based oral functional rehabilitation exercise content planned in this study was implemented smoothly in an actual mobile environment or whether elderly people could easily use it. Therefore, to develop an ICT-based oral functional rehabilitation exercise program that can apply this content, it is necessary to develop an application through collaboration with experts in various fields. In addition, it is believed that future research will require advancements in content through more detailed and sophisticated modifications and supplements. Despite these limitations, this study planned to develop evidence-based oral functional rehabilitation exercises through a systematic review and expert Delphi survey to improve and enhance oral function in elderly people, the importance of which has recently been emphasized. It is thought that its significance lies in the fact that we attempted to compose this into ICT-based content.

## 5. Conclusions

The importance of oral functional rehabilitation exercises to improve the oral function of elderly people is gradually increasing. However, very few studies have developed applied content by combining the concept of oral functional rehabilitation exercises with information and communication technology. This study’s development of information and communication technology-integrated content represents a significant step forward, offering a novel approach to improving the quality of life for this vulnerable population and potentially mitigating age-related oral-function decline. Nevertheless, the findings of this initial development study require validation through rigorous, large-scale clinical trials to confirm the long-term effectiveness and generalizability of the proposed program. Future research should prioritize such trials, incorporating objective measures of oral function and quality of life to assess the program’s true impact.

## Figures and Tables

**Table 1:** Current status of mobile application development related to oral function.

Operating System	Purpose	Application Name	Cost
IOS	Education	Smart Oral Motor	No
		Train My Tongue	No
	Health	Dysfagi træning	No
		Swallow RehApp	Yes
	Medical	Small Talk Oral Motor Exercises	No
Android	Lifestyle	Oral Health and Dementia	No
	Medical	Speech Companion	Yes
Both	Education	Oroeasy	No
		VAST Pre-Speech Motor Planning	Yes
		Tongue Thrust Therapy	No

**Table 2:** Detailed classification of OFRE content.

Intervention Domain		Detailed Content
Warm-up and cool-down exercise		Orofacial muscle-stretching exercise
Masticatory function exercise		Chewing exercise using noncariogenic foods or tools Masseter muscle-strengthening exercise
Swallowing function exercise	Tongue exercise	Tongue-strengthening exercise Swallowing exercise
	Pharyngeal exercise	Head-flexing exercise
	Lip and respiratory muscle exercise	Lip-strengthening exercise Expiratory muscle-strength exercise
Articulatory function exercise		Syllable-articulation exercise
Salivary function exercise		Salivary gland massage

**Table 3:** The final ICT-based OFRE contents.

Name	Menu	Contents	Details
OFRE	Oral-function exercise ability evaluation	Maximum mouth-opening measurement	Measure oral-function exercise ability before and after exercise (measure maximum opening amount)
	Oral Pilates	Warm-up exercise	Warm-up and cool-down are the same exercise
		Cool-down exercise	
		Masticatory function exercise	Eight exercises provided in video format
		Tongue exercise	
		Pharyngeal exercise	
		Lip exercise	
		Respiratory muscle exercise	
		Articulatory function exercise	
		Salivary function exercise	
	Game	Salivary secretion stimulation exercise	Perform oral functional rehabilitation exercises in a fun way like a motion recognition game.
		Lip and respiratory muscle exercise	
		Articulatory function exercise	
		Tongue and pharyngeal exercise	

## Data Availability

The data presented in this study are available on request from the corresponding author.

## References

[1] Bjering H., Curry J., Maeder A. (2014). Gerontechnology: The importance of user participation in ICT development for older adults. Investing in E-Health: People, Knowledge and Technology for a Healthy Future.

[2] Europe A.P. (2023). Certification for Ageing in Place: The New European Project “HOMES4LIFE” Kick Off. https://www.age-platform.eu/certification-for-ageing-in-place-the-new-european-project-homes4life-kicks-off/.

[3] Yoshimoto T., Nawa N., Uemura M., Sakano T., Fujiwara T. (2022). The impact of interprofessional communication through ICT on health outcomes of older adults receiving home care in Japan—A retrospective cohort study. J. Gen. Fam. Med..

[4] Waldman S.A., Terzic A. (2019). Healthcare evolves from reactive to proactive. Clin. Pharmacol. Ther..

[5] Ching Yuen Luk S. (2023). Technologies and the Wellness of Older Adults. Healthy Ageing in Singapore: Opportunities, Challenges and the Way Forward.

[6] Hänninen R., Taipale S., Luostari R. (2021). Exploring heterogeneous ICT use among older adults: The warm experts’ perspective. New Media Soc..

[7] Sheng Y., Doyle J., Bond R., Jaiswal R., Gavin S., Dinsmore J. (2022). Home-based digital health technologies for older adults to self-manage multiple chronic conditions: A data-informed analysis of user engagement from a longitudinal trial. Digit. Health.

[8] Yutong T., Yan Z., Qingyun C., Lixue M., Mengke G., Shanshan W. (2023). Information and Communication Technology Based Integrated Care for Older Adults: A Scoping Review. Int. J. Integr. Care.

[9] Kim J., Song J., Kim D., Park J. (2022). The development of ICT-based exercise rehabilitation service contents for patients with musculoskeletal disorders and stroke. Int. J. Environ. Res. Public Health.

[10] Barisch-Fritz B., Bezold J., Scharpf A., Trautwein S., Krell-Roesch J., Woll A. (2022). ICT-based individualized training of institutionalized individuals with dementia. Evaluation of usability and trends toward the effectiveness of the InCoPE-App. Front. Physiol..

[11] Nagatomi Y., Ide T., Higuchi T., Nezu T., Fujino T., Tohyama T., Nagata T., Higo T., Hashimoto T., Matsushima S. (2022). Home-based cardiac rehabilitation using information and communication technology for heart failure patients with frailty. ESC Heart Fail..

[12] Assis M.A.L., Tavares L.D.F., Bernardino A.P., Rocha B.A., Abreu L.G., Oliveira D.D., Pithon M.M., Soares R.V. (2022). Information and Communications Technology in Dentistry: An informative and educational approach for patients with fixed orthodontic appliances. Dental Press J. Orthod..

[13] Brenčič N.S., Muntianu L.S., Piotrowicz K., Mocanu I., Rudel D., Lupu I. Oral health education for elderly through ICT technology. Proceedings of the EDULEARN21 Proceedings.

[14] Hakeem F.F., Bernabe E., Sabbah W. (2019). Association between oral health and frailty: A systematic review of longitudinal studies. Gerodontology.

[15] Koyama Y., Sugimoto A., Hamano T., Kasahara T., Toyokura M., Masakado Y. (2017). Proposal for a modified jaw opening exercise for dysphagia: A randomized, controlled trial. Tokai J. Exp. Clin. Med..

[16] Iwao-Kawamura Y., Shigeishi H., Uchida S., Kawano S., Maehara T., Sugiyama M., Ohta K. (2021). Changes in physical and oral function after a long-term care prevention program in community-dwelling Japanese older adults: A 12-month follow-up study. Healthcare.

[17] Takano S., Yamaguchi K., Nakagawa K., Yoshimi K., Nakane A., Okumura T., Tohara H. (2021). Effect of isometric exercises on the masseter muscle in older adults with missing dentition: A randomized controlled trial. Sci. Rep..

[18] Kim M.J., Hong J.Y., Lee G., Yoon T., Hwang S.H., Kim H.H., Jung Y., Park J.S. (2020). Effects of chewing exercises on the occlusal force and masseter muscle thickness in community-dwelling Koreans aged 65 years and older: A randomised assessor-blind trial. J. Oral. Rehabil..

[19] Shirobe M., Watanabe Y., Tanaka T., Hirano H., Kikutani T., Nakajo K., Sato T., Furuya J., Minakuchi S., Iijima K. (2022). Effect of an oral frailty measures program on community-dwelling elderly people: A cluster-randomized controlled trial. Gerontology.

[20] Mansoor D.E. (2023). Full Mouth Rehabilitation versus Full Body Rehabilitation. https://www.fdiworlddental.org/full-mouth-rehabilitation-versus-full-body-rehabilitation.

[21] Choi Y., Yun J., Park H., Cha E. (2022). A Study on the Status of Contents Related to Oral Functional Rehabilitation Exercise Based on Application. J. Korean Soc. Oral. Healh Sci..

[22] Kwon H., Maeng H., Chung J. (2022). Development of an ICT-based exergame program for children with developmental disabilities. J. Clin. Med..

[23] Yun J.H. (2023). Development and Validation of a Conceptual Framework of ICF-Based Oral Function Rehabilitation Exercise Intervention for Community-Dwelling Older Adults. Ph.D. Thesis.

[24] Li X.-Y., Jia C., Zhang Z.-C. (2017). The normal range of maximum mouth opening and its correlation with height or weight in the young adult Chinese population. J. Dent. Sci..

[25] Peel N.M., McClure R.J., Bartlett H.P. (2005). Behavioral determinants of healthy aging. Am. J. Prev. Med..

[26] Haumschild M.S., Haumschild R.J. (2009). The importance of oral health in long-term care. J. Am. Med. Dir. Assoc..

[27] Suzuki M., Koyama S., Kimura Y., Ishiyama D., Otobe Y., Nishio N., Ichikawa T., Kunieda Y., Ohji S., Ito D. (2018). Relationship between characteristics of skeletal muscle and oral function in community-dwelling older women. Arch. Gerontol. Geriatr..

[28] Watanabe Y., Hirano H., Arai H., Morishita S., Ohara Y., Edahiro A., Murakami M., Shimada H., Kikutani T., Suzuki T. (2017). Relationship between frailty and oral function in community-dwelling elderly adults. J. Am. Geriatr. Soc..

[29] Kobayashi M., Hiyama A., Miura T., Asakawa C., Hirose M., Ifukube T. Elderly user evaluation of mobile touchscreen interactions. Proceedings of the Human-Computer Interaction—INTERACT.

[30] Mariño R.J., Marwaha P., Barrow S.-y. (2016). Web-based oral health promotion program for older adults: Development and preliminary evaluation. Int. J. Med. Inform..

[31] Wongworasun S., Hunsrisakhun J., Watanapa A. (2022). Effectiveness of oral exercise programs on oral function among independent elderly people: A cluster randomized controlled trial. J. Int. Oral Health.

[32] Lee J., Kwon H., Lee Y., Lee M.H., Lee H.K. (2010). Effect of regular oral exercise on oral function in elderly patients with long-term care. J. Korean Acad. Oral. Health.

[33] Jang K., Hwang I. (2011). Effects of mouth exercise on the improvements of oral function in elderly men. J. Dent. Hyg. Sci..

[34] Lee J.W., Park S.J. (2013). A study on interface design of serious game for the elderly. Adv. Sci. Technol. Lett..

[35] Resnick B., Vogel A., Luisi D. (2006). Motivating minority older adults to exercise. Cultur. Divers. Ethnic Minor. Psychol..

[36] Ki J.Y., Jo S.R., Cho K.S., Park J.E., Cho J.W., Jang J.H. (2021). Effect of oral health education using a mobile app (OHEMA) on the oral health and swallowing-related quality of life in community-based integrated care of the elderly: A randomized clinical trial. Int. J. Environ. Res. Public Health.

[37] Ohara Y., Yoshida N., Kono Y., Hirano H., Yoshida H., Mataki S., Sugimoto K. (2015). Effectiveness of an oral health educational program on community-dwelling older people with xerostomia. Geriatr. Gerontol. Int..

[38] Sánchez-Mena A., Martí-Parreño J. (2017). Drivers and barriers to adopting gamification: Teachers’ perspectives. Electron. J. e-Learn..

[39] Kim d. (2020). A Literature review of domestic research on gamification of education in Korea: Research trends and critical analysis. Korean Assoc. Learn.-Centered Curric. Instr..

[40] Johnson L., Becker S.A., Estrada V., Freeman A. (2014). NMC Horizon Report: 2014 K-12 Edition. The New Media Consortium. https://files.eric.ed.gov/fulltext/ED559369.pdf.

